# Supplementary material for: Effects of sex and site on amino acid metabolism enzyme gene expression and activity in rat white adipose tissue
Source: PeerJ. 2015 Nov 10;3:e1399. doi: 10.7717/peerj.1399 (PMC4647552; doi:10.7717/peerj.1399)
Supplement: Supplemental Information 1 [file peerj-03-1399-s001.docx]

**SUPPLEMENTAL MATERIAL**

SUPPLEMENTAL METHODS

**Measurement of urea cycle enzyme activities**

Ornithine carbamoyl transferase activity was measured from the reaction of condensation of carbamoyl-P and 14C-ornithine to yield 14C-citrulline. Aliquots of 25 μL of homogenates were mixed with 50 μL of 70 mM hepes buffer pH 7.4 containing carbamoyl-P, ornithine (all from Sigma), and 14C-ornithine (Perkin-Elmer); final concentrations were 9 mM, 13 mM and 1 kBq/mL, respectively. The reaction was started with the homogenate, and was carried out at 37ºC. Aliquots of 75 μL were introduced in tubes containing 100 μL of chilled acetone, in ice, at 0, 0.5,1 and 2 min. The tubes were later centrifuged to obtain clear supernatants; they were dried in a vacuum-centrifuge (Thermo Scientific, Waltham, MA USA). The dry residues were dissolved in 25 μL of pure water; they were applied to silicagel TLC plates (200 µm; Macherey-Nagel, Düren, Germany), etched vertically to define 1 cm wide lanes to prevent sample overlapping. Standards of ornithine and citrulline were included each plate. The plates were developed with a mobile phase of trichloromethane: methanol: acetic acid (1:2:2 by volume). After drying, the lane with non-labelled standards was sprayed with ninhydrin, and heated with a hair dryer. The ornithine and citrulline spots were marked and the plate was cut in horizontal zones, which were counted. To limit interferences , the label in the citrulline area was expressed as a percentage of the total label counted in each TLC lane. These data allowed the calculation of newly formed citrulline; the Vi value for each sample was plotted, and considered to be an expression of V_max_ under the conditions tested.

Arginino-succinate synthase activity was measured from the condensation of aspartate with citrulline in the presence of ATP to yield argininosuccinate. Aliquots of 55 μL of homogenates were mixed with 30 μL of 70 mM hepes buffer pH 7.4, containing ATP-Na_2_, MgCl_2_, citrulline, aspartate (all from Sigma); final concentrations were 10 mM, 5 mM, 3 mM, and 2.5 mM, respectively. The reaction was started with aspartate, and was carried out at 37ºC. The reaction was stopped with 40 μL of 30 g/L perchloric acid. The tubes were vortexed and immediately neutralized (pH 7-8) with 10 μL of 100 g/L KOH containing 62 g/L potassium bicarbonate. The tubes were vortexed again and centrifuged at 8,000xg for 15 min and 4ºC. The aspartate remaining in the supernatants was measured (by transamination to oxaloacetate, and then reduced by malate dehydrogenase and NADH). Briefly, 20 μL of the supernatants were brought up to 300 μL in 96-well plates, with 66 mM phosphate buffer pH 7.4 containing NADH, 2-oxoglurarate, aspartate transaminase (pig heart) and malic acid dehydrogenase (pig heart) (all from Sigma); final concentrations were, respectively, 0.25 mM, 0.2 mM, 20 nkat/ml and 17 nkat/ml. The samples were read at 340 nM in a plate reader (Biotek, Winoosky, VT USA) for 30 min at intervals of 30 s. The fall in NADH was used to determine the levels of aspartate at each incubation time. Its disappearance (versus time zero levels) was used to determine the amount of aspartate incorporated into argininosuccinate. As in the case of ornithine carbamoyl transferase, the Vi values were plotted for each sample. In all series, a number of samples were analyzed with all reagents except citrulline: No spurious consumption of aspartate was observed

The activity of argininosuccinate synthase was measured from the breakup of argininosuccinate to yield fumarate and arginine. This amino acid was measured in a second reaction using arginase to form ornithine and urea, which was analyzed using a chemical method. Aliquots of 38 μL of homogenates were mixed with 38 μL of 70 mM hepes buffer pH 7.4, containing argininosuccinate (Sigma); final concentration 2 mM. Incubations were carried out at 37ºC for 0, 2.5, 5 and 10 min. The reaction was stopped by the addition of 40 μL of 30 g/L perchloric acid. The tubes were vortexed and brought to pH 8-9 with 10 μL of 100 g/L KOH containing 80 g/L potassium bicarbonate. The tubes were mixed again, and centrifuged for 15 min in the cold (4ºC) at 8,000xg. Aliquots of 100 μL of the supernatants were mixed with 50 μL of the reacting mixture: 70 mM hepes buffer pH 7.5 (to achieve a final pH 8.5) containing MnCl_2_ and arginase (rat liver, Lee Biosolutions, St Louis, MO USA); final concentrations 7 mM and 17 nkat/mL. The buffer containing Mn and arginase was activated (heated) for 5 min at 55ºC before use. The reaction was allowed to develop for 30 min at 37ºC, and was stopped by the addition of 35 μL 160 g/L perchloric acid. The tubes were centrifuged for 15 min at 8,000xg and 4ºC. The acidic supernatants (175 μL) were used for the estimation of urea. They were mixed with 300 μL of 90 g/L H_2_SO_4_ containing 270 g/L H_3_PO_4_; then 20 μL of 30 g/L of 1-phenyl-2-oxime-1,2-propanodione (Sigma) in absolute ethanol were added (MC). The reaction was developed at 100ºC for 30 min in a dry block heater. The absorbance of the tubes was measured at 540 nm using a plate reader, including standards of arginine and urea as well as blanks. Under these conditions, conversion of arginine to urea was complete.

Total arginase activity was measured through the estimation of the urea produced from arginine in the presence of Mn^2^+ (MG). Aliquots of 20 μL of homogenates were mixed with 5 μL of 50 mM MnCl_2_. The tubes were heated for 5 min at 55ºC to activate arginase (MH). Then, the temperature was brought down to 37ºC. The reaction began with the addition of 75 μL of arginine (Sigma); final concentration 78 mM. Incubations were carried out for 1, 10 and 20 min at 37ºC. The reaction was stopped by the addition of 35 μL 160 g/L perchloric acid. The tubes were centrifuged at 8,000xg and 4ºC for 15 min. The measurement of urea generated was done as described above for argininosuccinate lyase. Since the method used for arginase does not differentiate between arginases 1 and 2, we present a combined total arginase value that corresponds mostly to arginase 1 (expressed in all WAT sites, whilst arginase 2 was expressed only in SC WAT).

SUPPLEMENTAL TABLES

**Supplemental Table 1** Urea cycle enzyme activities in WAT sites of female and male Wistar rats

| enzyme | units | sex | SC | ME | RP | PG | P (site) | P (sex) |
| --- | --- | --- | --- | --- | --- | --- | --- | --- |
| glutamine-dependent carbamoyl-phosphate synthase | nkat/gP | M | 165 ± 35* | 49.0 ± 13.7 | 56.5 ± 9.2 | 40.4 ± 9.3 | <0.0001 | NS |
|  |  | F | 88.0 ± 30.8 | 55.3 ± 6.7 | 61.6 ± 9.9 | 53.8 ± 13.3 |  |  |
| ornithine carbamoyl transferase |  | M | 2434 ± 1108 | 2792 ± 872 | 3206 ± 403 | 1975 ± 496 | NS | NS |
|  |  | F | 3871 ± 981 | 2051 ± 598 | 3015 ± 579 | 2585 ± 727 |  |  |
| argininosuccinate synthase |  | M | 82 ± 11.4 | 57.3 ± 8.5 | 73.8 ± 15.3 | 87.8 ± 17.7 | NS | NS |
|  |  | F | 51.7 ± 7.5 | 114 ± 15.4* | 40.7 ± 13.1 | 61.2 ± 9.0 |  |  |
| argininosuccinate lyase |  | M | 291 ± 40 | 170 ± 33 | 90.1 ± 6.7 | 147 ± 35 | <0.0001 | NS |
|  |  | F | 501 ± 108* | 166 ± 37 | 79.8 ± 10.3 | 162 ± 42.3 |  |  |
| arginase (total) |  | M | 435 ± 103 | 220 ± 50 | 297 ± 168 | 361 ± 117 | NS | NS |
|  |  | F | 316 ± 82 | 232 ± 32 | 335 ± 64 | 337 ± 51 |  |  |

The data are the mean ± sem of 6 animals per group. Statistical significance of the differences between groups was established with a 2-way anova; post-hoc Tuckey test: different superscript letters represent P<0.05 differences

**Supplemental Table 2** Urea cycle enzyme gene expressions in WAT sites of female and male Wistar rats

| enzyme (gene) | units | sex | SC | ME | RP | PG | P (site) | P (sex) |
| --- | --- | --- | --- | --- | --- | --- | --- | --- |
| glutamine-dependent carbamoyl-phosphate synthase, type 2 (*Cad*) | fmol/gP | M | 58.3 ± 7.9 | 56.3 ± 6.3 | 12.8 ± 1.9 | 43.0 ± 4.2 | <0.0001 | 0.0002 |
|  |  | F | 95.6 ± 4.7* | 63.4 ± 6.1 | 24.5 ± 5.9 | 55.0 ± 5.9 |  |  |
| ornithine carbamoyltransferase (*Otc*) |  | M | 2.99 ± 0.95 | 0.19 ± 0.02 | 0.76 ± 0.32 | 0.91 ± 0.13 | 0.008 | NS |
|  |  | F | 1.17 ± 0.07 | 0.23 ± 0.07 | 1.35 ± 0.57 | 2.19 ± 0.94 |  |  |
| argininosuccinate synthase 1(*Ass1*) |  | M | 10.2 ± 1.8 | 4.68 ± 0.61 | 3.62 ± 1.12 | 5.02 ± 0.35 | NS | NS |
|  |  | F | 6.39 ± 0.71 | 5.96 ± 1.01 | 5.58 ± 1.84 | 13.4 ± 5.4* |  |  |
| argininosuccinate lyase (*Asl*) |  | M | 57.1 ± 8.4 | 27.3 ± 5.7 | 10.3 ± 2.4 | 36.0 ± 4.4 | <0.0001 | NS |
|  |  | F | 44.9 ± 2.4 | 34.5 ± 4.9 | 14.8 ± 1.5 | 28.0 ± 2.8 |  |  |
| arginase (*Arg1* + *Arg2)* |  | M | 21.2 ± 1.7* | 0.74 ± 0.24 | 0.39 ± 0.15 | 2.74 ± 0.56 | <0.0001 | NS |
|  |  | F | 16.1 ± 2.1 | 0.41 ± 0.04 | 1.59 ± 0.50 | 3.23 ± 0.67 |  |  |

The data are the mean ± sem of 6 animals per group. Statistical significance of the differences between groups was established with a 2-way anova; post-hoc Tuckey test: different superscript letters represent P<0.05 differences: "Total" arginase is the composite value of the sums of expressions of the genes for arginases 1 and 2, affecting only SC data, since in the other sites, only arginase 1 was detected. The separate data for arginases is shown in Supplemental Table 3.

**Supplemental Table 3** Amino acid metabolism enzyme gene expressions in WAT sites of female and male Wistar rats

| enzyme (gene) | sex | SC | ME | RP | PG | P (site) | P (sex) |
| --- | --- | --- | --- | --- | --- | --- | --- |
| N-acetylglutamate synthase (*Nags*) | M | 1.13 ± 0.31 | 0.85 ± 0.32 | 0.43 ± 0.04 | 0.96 ± 0.15 | NS | NS |
|  | F | 0.88 ± 0.25 | 1.04 ± 0.33 | 0.97 ± 0.08 | 1.21 ± 0.32 |  |  |
| arginase-1 (*Arg1)* | M | 3.29 ± 1.08 | 0.74 ± 0.24 | 0.39 ± 0.15 | 2.74 ± 0.56 | 0.0042 | NS |
|  | F | 4.25 ± 1.38 | 0.41 ± 0.04 | 1.59 ± 0.50 | 3.23 ± 0.67 |  |  |
| arginase-2, mitochondrial (*Arg2*) | M | 17.9 ± 2.4 | -- | -- | -- | -- | -- |
|  | F | 11.1 ± 2.7 | -- | -- | -- |  |  |
| nitric oxide synthase, endothelial (*Nos3*) | M | 75.4 ± 9.5* | 21.6 ± 2.6 | 14.6 ± 1.1 | 31.6 ± 6.3 | <0.0001 | 0.0031 |
|  | F | 43.2 ± 2.8 | 18.5 ± 1.0 | 16.1 ± 2.2 | 22.6 ± 2.4 |  |  |
| glutamine synthetase (*Glul*) | M | 1336 ± 523* | 198 ± 29 | 136 ± 9 | 426 ± 99 | 0.0005 | NS |
|  | F | 461 ± 49 | 225 ± 38 | 186 ± 39 | 308 ± 42 |  |  |
| glutaminase kidney isoform, mitochondrial (*Gls*) | M | 39.9 ± 4.8 | 20.1 ± 1.5 | 15.4 ± 1.1 | 24.2 ± 2.7 | <0.0001 | <0.0001 |
|  | F | 77.5 ± 6.4* | 24.4 ± 2.9 | 22.2 ± 2.7 | 23.6 ± 3.6 |  |  |
| glutamate dehydrogenase 1, mitochondrial (*Glud1*) | M | 451 ± 80 | 410 ± 62 | 119 ± 13 | 272 ± 32 | <0.0001 | NS |
|  | F | 350 ± 37 | 454 ± 62 | 170 ± 13 | 300 ± 11 |  |  |
| glycine cleavage system H protein, mitochondrial (*Gcsh*) | M | 110 ± 15 | 60.6 ± 11.8 | 45.8 ± 3.9 | 72.9 ± 12.2 | NS | NS |
|  | F | 109 ± 31 | 62.8 ± 7.8 | 86.1 ± 15.2 | 90.1 ± 5.2 |  |  |
| AMP deaminase 2 (*Ampd2*) | M | 125 ± 21* | 56.4 ± 14.6 | 14.5 ± 1.3 | 32.9 ± 3.6 | <0.0001 | NS |
|  | F | 73.5 ± 6.7 | 83.9 ± 9.9 | 22.0 ± 2.4 | 32.4 ± 2.7 |  |  |
| alanine aminotransferase 1 (*Gpt*) | M | 434 ± 104* | 156 ± 53 | 115 ± 10 | 325 ± 41 | 0.0033 | NS |
|  | F | 193 ± 31 | 191 ± 49 | 163 ± 31 | 268 ± 39 |  |  |
| alanine aminotransferase 2 (*Gpt2*) | M | 10.1 ± 2.2 | 4.76 ± 1.68 | 4.52 ± 0.69 | 21.7 ± 3.7* | <0.0001 | 0.0018 |
|  | F | 6.72 ± 1.02 | 3.86 ± 0.97 | 4.15 ± 1.00 | 9.03 ± 1.59 |  |  |
| branched-chain-amino-acid aminotransferase, cytosolic (*Bcat1*) | M | 13.5 ± 1.8 | 14.9 ± 0.9 | 3.15 ± 0.43 | 12.7 ± 2.5 | <0.0001 | NS |
|  | F | 13.6 ± 3.0 | 14.4 ± 3.3 | 3.20 ± 0.42 | 8.31 ± 0.95 |  |  |
| branched-chain-amino-acid aminotransferase, mitochondrial (*Bcat2*) | M | 176 ± 49 | 132 ± 33 | 56.7 ± 5.0 | 142 ± 37 | 0.0344 | NS |
|  | F | 113 ± 18 | 114 ± 20 | 80.5 ± 6.3 | 142 ± 7 |  |  |

The data are the mean ± sem of 6 animals per group, and are expressed as fkat/g of protein. Statistical significance of the differences between groups was established with a 2-way anova; post-hoc Tuckey test: different superscript letters represent P<0.05 differences. The data for arginase 2 were compared with an 1-way anova test and pos

**Supplemental Table 4** Lipid metabolism-related protein gene expressions in WAT sites of female and male Wistar rats

| protein (gene) | sex | SC | ME | RP | PG | P (site) | P (sex) |
| --- | --- | --- | --- | --- | --- | --- | --- |
| solute carrier family 2 (facilitated glucose transporter), member 4 | M | 108 ± 29 | 31.2 ± 8.6 | 44.0 ± 6.5 | 109 ±8.1 | NS | 0.0040 |
|  | F | 160 ± 18 | 116 ± 36 | 119 ± 43 | 160 ± 54 |  |  |
| hexokinase-2 (*Hk2*) | M | 11.7 ± 2.5 | 7.89 ± 1.18 | 4.12 ± 0.39 | 16.9 ± 2.8 | 0.0057 | 0.0009 |
|  | F | 18.9 ± 3.7 | 16.8 ± 3.2 | 13.5 ± 4.5 | 25.6 ± 5.7 |  |  |
| glucose-6-phosphate 1- dehydrogenase (*G6pdx*) | M | 334 ± 73 | 129 ± 18 | 84.2 ± 14.2 | 129 ± 12 | 0.0005 | 0.0196 |
|  | F | 267 ± 25 | 202 ± 32 | 183 ± 35 | 285 ± 51* |  |  |
| NADP-dependent malic enzyme (*Me1*) | M | 1672 ± 684 | 47.3 ± 13 | 110 ± 27 | 120 ± 28 | NS | 0.0244 |
|  | F | 725 ± 200 | 1251 ± 379* | 1361 ± 307* | 721 ± 255 |  |  |
| pyruvate dehydrogenase kinase 2, mitochondrial (*Pdk2*) | M | 269 ± 87 | 120 ± 24 | 48.6 ± 7.6 | 91.7 ± 12.8 | 0.0013 | NS |
|  | F | 146 ± 13 | 193 ± 51 | 63.9 ± 10.4 | 86.8 ± 16.8 |  |  |
| pyruvate dehydrogenase [acetyl transferring] kinase 4, mitochondrial (*Pdk4*) | M | 180 ± 73* | 14.2 ± 3.7 | 13.5 ± 3.5 | 14.5 ± 5.4 | 0.0016 | 0.0203 |
|  | F | 22.7 ± 2.6 | 7.64 ± 3.86 | 6.10 ± 1.00 | 8.52 ± 3.29 |  |  |
| ATP citrate lyase (*Acly*) | M | 167 ± 81 | 22.2 ± 2.9 | 25.1 ± 4.7 | 109 ± 10 | NS | 0.0100 |
|  | F | 186 ± 54 | 160 ± 34 | 295 ± 140* | 228 ± 102 |  |  |
| acetyl-CoA carboxylase 1 (*Acaca*) | M | 74.0 ± 16.3 | 30.6 ± 3.9 | 81.0 ± 7.2 | 126 ± 12 | 0.0038 | 0.0035 |
|  | F | 149 ± 37 | 165 ± 49 | 542 ± 206* | 156 ± 66 |  |  |
| fatty acid synthase (*Fasn*) | M | 7620 ± 4114 | 191 ± 74 | 572 ± 85 | 1269 ± 250 | NS | NS |
|  | F | 2447 ± 403 | 1657 ± 536 | 3318 ± 1130 | 2106 ± 795 |  |  |
| carnitine palmitoyltransferase 1, liver isoform (*Cpt1a*) | M | 39.6 ± 14.8* | 14.0 ± 2.7 | 3.31 ± 0.51 | 16.3 ± 1.7 | 0.0002 | NS |
|  | F | 19.4 ± 3.9 | 13.7 ± 3.6 | 2.02 ± 0.36 | 10.8 ± 1.7 |  |  |
| carnitine palmitoyltransferase 2, mitochondrial (*Cpt2*) | M | 10.6 ± 3.0 | 10.7 ± 1.5 | 13.4 ± 1.7 | 25.1 ± 4.3 | 0.0150 | NS |
|  | F | 17.0 ± 1.8 | 14.9 ± 3.6 | 20.3 ± 3.4 | 19.2 ± 3.4 |  |  |
| long-chain acyl-CoA dehydrogenase, mitochondrial (*Acadl*) | M | 1076 ± 347* | 166 ± 9 | 204 ± 22 | 353 ± 72 | 0.022 | NS |
|  | F | 365 ± 23 | 298 ± 60 | 302 ± 49 | 412 ± 91 |  |  |
| adipose triglyceride lipase (*Atgl*) | M | 1322 ± 297* | 429 ± 95 | 700 ± 48 | 1453 ± 296 | 0.0002 | 0.0425 |
|  | F | 701 ± 26 | 338 ± 95 | 732 ± 51 | 1044 ± 149 |  |  |
| hormone-sensitive lipase (*Lipe*) | M | 631 ± 131 | 166 ± 38 | 458 ± 39 | 674 ± 59 | <0.0001 | NS |
|  | F | 486 ± 37 | 218 ± 61 | 645 ± 84 | 664 ± 64 |  |  |
| lipoprotein lipase (*Lpl*) | M | 6299 ± 1305* | 1192 ± 279 | 2283 ± 139 | 3969 ± 719 | <0.0001 | NS |
|  | F | 3788 ± 444 | 1097 ± 80 | 2783 ± 588 | 4316 ± 294 |  |  |

The data are the mean ± sem of 6 animals per group, and are expressed as fkat/g of protein . Statistical significance of the differences between groups was established with a 2-way anova; post-hoc Tuckey test: different superscript letters represent P<0.05 differences.

**Supplemental Table 5** Comparison of male-female specific expression of genes in different WAT sites with respect to tissue total RNA.

| gene | sex | SC | | ME | | PG | | RP | | | all-sites P value |
| --- | --- | --- | --- | --- | --- | --- | --- | --- | --- | --- | --- |
|  |  | fmol/mgRNA | P | fmol/mgRNA | P | fmol/mgRNA | P | fmol/mgRNA | | P |  |
| ***urea cycle enzymes*** | | | | | | | | | | | |
| ornithine carbamoyl-transferase | M | 0.61 ± 0.16 | NS | 0.020 ± 0.003 | NS | 0.42 ± 0.05 | NS | 1.08 ± 0.46 | | NS | NS |
|  | F | 0.27 ± 0.01 |  | 0.031 ± 0.007 |  | 1.03 ± 0.43 |  | 1.24 ± 0.54 | |  |  |
| carbamoyl-P synthase 2 | M | 17.0 ± 2.0 | 0.0220 | 6.19 ± 0.27 | NS | 16.8 ± 2.7 | NS | 16.4± 1.4 | | NS | NS |
|  | F | 24.0 ± 1.6 |  | 5.87 ± 0.54 |  | 17.3 ± 2.4 |  | 18.4 ± 3.2 | |  |  |
| arginino-succinate synthase | M | 1.94 ± 0.19 | NS | 0.51 ± 0.04 | NS | 2.69 ± 0.21 | NS | 3,67 ± 1.11 | | NS | NS |
|  | F | 1.51 ± 0.18 |  | 0.62 ± 0.11 |  | 6.31 ± 2.45 |  | 4.31 ± 1.51 | |  |  |
| arginino-succinate lyase | M | 13.8 ± 1.2 | NS | 3.07 ± 0.45 | NS | 18.5 ± 3.2 | NS | 11.2 ± 1.1 | | NS | NS |
|  | F | 10.6 ± 0.7 |  | 3.65 ± 0.37 |  | 15.1 ± 2.0 |  | 12.1 ± 1.4 | |  |  |
| arginase 1 | M | 0.56 ± 0.12 | NS | 0.065 ± 0.018 | NS | 1.28 ± 0.25 | NS | 0.50 ± 0.17 | | NS | NS |
|  | F | 0.93 ± 0.28 |  | 0.045 ± 0.010 |  | 1.60 ± 0.38 |  | 1.43 ± 0.61 | |  |  |
| ***Other enzymes of amino acid metabolism*** | | | | | | | | | | | |
| N-acetyl-glutamate synthase | M | 0.44 ± 0.095 | 0.0197 | 0.11 ± 0.035 | NS | 0.60 ± 0.18 | NS | | 0.59 ± 0.05 | NS | NS |
|  | F | 0.12 ± 0.01 |  | 0.12 ± 0.037 |  | 0.62 ± 0.15 |  |  | 0.72 ± 0.14 |  |  |
| glutamate dehydrogenase 1 | M | 112 ± 17 | NS | 38.9 ± 3.6 | NS | 142 ± 11 | NS | | 155 ± 9 | NS | NS |
|  | F | 82.9 ± 10 |  | 48.4 ± 6.1 |  | 157 ± 14 |  |  | 134 ± 16 |  |  |
| glutamine synthetase | M | 291 ± 74 | NS | 19.0 ± 2.0 | NS | 166 ± 9.9 | NS | | 178 ± 5 | NS | 0.0035 |
|  | F | 109 ± 11 |  | 20.0 ± 1.3 |  | 139 ± 15 |  |  | 139 ± 19 |  |  |
| glutaminase | M | 10.5 ± 1.9 | NS | 1.81 ± 0.22 | 0.0292 | 12.0 ± 1.0 | NS | | 20.4 ± 0.8 | NS | NS |
|  | F | 16.4 ± 2.5 |  | 2.62 ± 0.23 |  | 10.8± 1.5 |  |  | 17.2 ± 1.6 |  |  |
| AMP deaminase | M | 25.6 ± 2.3 | 0.0109 | 9.05 ± 0.79 | NS | 16.9± 1.2 | NS | | 19.1 ± 1.2 | NS | 0.0048 |
|  | F | 16.8 ± 1.4 |  | 8.87 ± 0.41 |  | 16.0± 0.9 |  |  | 17.8 ± 1.5 |  |  |
| glycine cleavage system | M | 26.8 ± 4.0 | NS | 7.08 ± 1.22 | NS | 32.8 ± 3.2 | 0.0128 | | 61.3 ± 5.4 | NS | NS |
|  | F | 18.4 ± 3.0 |  | 6.64 ± 0.82 |  | 45.7 ± 2.8 |  |  | 65.2 ± 6.4 |  |  |
| alanine transaminase 1 | M | 91.7 ± 6.3 | 0.0007 | 16.5 ± 5.1 | NS | 150 ± 9 | NS | | 152 ± 10 | 0.0409 | <0.0001 |
|  | F | 45.3 ± 7.1 |  | 16.3 ± 3.6 |  | 121 ± 15 |  |  | 114 ± 13 |  |  |
| alanine transaminase 2 | M | 2.67 ± 0.27 | 0.0104 | 0.49 ± 0.15 | NS | 9.90 ± 1.22 | 0.0034 | | 6.00 ± 0.68 | 0.0057 | <0.0001 |
|  | F | 1.58 ± 0.21 |  | 0.56 ± 0.18 |  | 3.99 ± 0.46 |  |  | 3.05 ± 0.42 |  |  |
| branched-chain amino acid transaminase 1 | M | 3.07 ± 0.41 | NS | 1.62 ± 0.13 | NS | 4.90 ± 0.25 | 0.0419 | | 4.04 ± 0.37 | 0.0087 | 0.0110 |
|  | F | 3.15 ± 0.59 |  | 1.50 ± 0.20 |  | 3.82 ± 0.42 |  |  | 2.54 ± 0.25 |  |  |
| branched-chain amino acid transaminase 2 | M | 36.5 ± 9.8 | NS | 15.6 ± 3.0 | NS | 53.5 ± 5.8 | NS | | 74.9 ± 6.0 | NS | NS |
|  | F | 26.1 ± 3.6 |  | 14.6 ± 2.4 |  | 66.0 ± 4.7 |  |  | 65.5 ± 4.3 |  |  |
| endothelial nitric oxide synthase | M | 22.8 ± 4.1 | 0.0382 | 2.13 ± 0.28 | NS | 14.3 ± 1.9 | NS | | 19.7 ± 1.9 | 0.0102 | 0.0001 |
|  | F | 11.4 ± 1.5 |  | 2.18 ± 0.29 |  | 10.4 ± 1.1 |  |  | 12.4 ± 1.0 |  |  |

(**Continues in next page**)

)

| gene | sex | SC | | | ME | | PG | | RP | | all-sites P value |
| --- | --- | --- | --- | --- | --- | --- | --- | --- | --- | --- | --- |
|  |  | fmol/mgRNA | P | | fmol/mgRNA | P | fmol/mgRNA | P | fmol/mgRNA | P |  |
| ***enzymes (and transporters) related with lipogenesis from glucose*** | | | | | | | | | | | |
| glucose transporter 4 | M | 36.1 ± 7.9 | | NS | 4.31 ± 1.43 | NS | 47.3 ± 6.8 | NS | 56.8 ± 5.6 | 0.0432 | 0.0115 |
|  | F | 46.7 ± 9.5 | |  | 9.34 ± 2.48 |  | 69.6± 21.2 |  | 101 ± 16 |  |  |
| hexokinase 2 | M | 3.02 ± 0.18 | | NS | 1.36 ± 0.31 | NS | 7.07 ± 1.05 | 0.0365 | 5.49 ± 0.56 | 0.0392 | 0.0001 |
|  | F | 4.45 ± 0.79 | |  | 2.26 ± 0.46 |  | 12.8 ± 2.0 |  | 11.5 ± 2.2 |  |  |
| glucose-6P dehydrogenase | M | 72.7 ± 4.0 | | NS | 13.1 ± 2.3 | 0.0458 | 61.9 ± 7.8 | 0.0192 | 108 ± 10 | 0.0198 | 0.0001 |
|  | F | 63.8 ± 6.7 | |  | 21.6 ± 2.9 |  | 128 ± 20 |  | 197 ± 27 |  |  |
| malic enzyme | M | 344 ± 103 | | NS | 5.15 ± 1.02 | 0.0485 | 65.5 ± 14.0 | 0.0476 | 147 ± 24 | 0.0218 | 0.0018 |
|  | F | 170 ± 43 | |  | 81.7 ± 29.5 |  | 313 ± 95 |  | 1022 ± 267 |  |  |
| pyruvate dehydrogenase kinase 2 | M | 52.7 ± 7.1 | | 0.0450 | 14.4 ± 2.6 | NS | 39.3 ± 4.4 | NS | 56.2 ± 2.9 | 0.0458 | 0.0319 |
|  | F | 33.9 ± 2.1 | |  | 15.6 ± 3.0 |  | 38.7 ± 6.3 |  | 47.8 ± 2.3 |  |  |
| pyruvate dehydrogenase kinase 4 | M | 37.2 ± 9.0 | | 0.0164 | 1.49 ± 0.30 | NS | 6.49 ± 1.48 | NS | 18.8 ± 5.2 | 0.0458 | <0.0001 |
|  | F | 5.35 ± 0.72 | |  | 0.93 ± 0.36 |  | 3.89 ± 1.24 |  | 5.10 ± 1.09 |  |  |
| citrate: ATP lyase | M | 33.4 ± 10.6 | | NS | 4.58 ± 1.07 | 0.0045 | 48.5 ± 9.8 | NS | 36.3 ± 8.5 | 0.0384 | 0.0016 |
|  | F | 46.0 ± 12.3 | |  | 28.0 ± 4.9 |  | 107 ± 43 |  | 290 ± 91 |  |  |
| acetyl-CoA carboxylase | M | 21.2 ± 3.6 | | 0.0442 | 3.39 ± 0.51 | 0.0242 | 55.0 ± 10.3 | NS | 112 ± 5 | 0.0100 | <0.0001 |
|  | F | 58.2 ± 13.9 | |  | 24.1 ± 6.5 |  | 120 ± 39 |  | 466 ± 88 |  |  |
| fatty acid synthase | M | 936 ± 234 | | NS | 39.5 ± 14.7 | 0.0143 | 496 ± 98 | 0.0483 | 789 ± 85 | 0.0348 | 0.0018 |
|  | F | 696 ± 134 | |  | 157 ± 33 |  | 1553 ±408 |  | 2286 ± 523 |  |  |
| ***enzymes (and transporters) related with lipolysis and fatty acid oxidation*** | | | | | | | | | | | |
| carnitine palmitoleoyl transferase (liver) | M | 11.7 ± 2.8 | 0.0491 | | 1.88 ± 0.23 | NS | 8.90 ± 1.09 | 0.0137 | 4.23 ± 0.43 | 0.001 | <0.0001 |
|  | F | 4.55 ± 0.83 |  |  | 1.33 ± 0.18 |  | 4.92 ± 0.66 |  | 1.68 ± 0.33 |  |  |
| carnitine palmitoleoyl transferase (muscle) | M | 3.75 ± 0.91 | NS | | 1.18 ± 0.17 | NS | 11.7 ± 1.9 | NS | 19.9 ± 2.1 | NS | 0.0350 |
|  | F | 3.49 ± 0.60 |  |  | 1.55 ± 0.35 |  | 8.67 ± 1.18 |  | 15.2 ± 1.2 |  |  |
| long-chain acyl-CoA dehydrogenase | M | 210 ± 38 | 0.0214 | | 18.5 ± 1.8 | 0.0462 | 168 ± 24 | NS | 271 ± 26 | NS | 0.0460 |
|  | F | 86.0 ± 5.5 |  |  | 30.9 ± 4.7 |  | 185 ± 37 |  | 229 ± 16 |  |  |
| adipose tissue triacylglycerol lipase | M | 337 ± 73 | 0.0460 | | 43.2 ± 9.7 | NS | 667 ± 105 | NS | 929 ± 61 | 0.0017 | <0.0001 |
|  | F | 145 ± 22 |  |  | 34.3 ± 7.2 |  | 477 ± 66 |  | 577 ± 56 |  |  |
| hormone-sensitive lipase | M | 168 ± 37 | NS | | 16.7 ± 3.9 | NS | 379 ± 48 | NS | 603 ± 32 | NS | 0.0131 |
|  | F | 114 ± 8 |  |  | 17.5 ± 4.0 |  | 306 ± 31 |  | 499 ± 46 |  |  |
| lipoprotein lipase | M | 1553 ± 68 | 0.0324 | | 154 ± 41 | NS | 1796 ± 211 | NS | 3458 ± 372 | 0.0440 | NS |
|  | F | 1038 ± 177 |  |  | 132 ± 28 |  | 2269 ± 263 |  | 2275 ± 354 |  |  |

**Supplemental Table 5 (conclusion)**

The data are the mean ± sem of 6 animals per group, and are expressed as fmol of the corresponding mRNA per mg of total RNA. Statistical significance of the differences between groups was established with 1- and 2-way anova; NS = P>0.05.
